# A Novel Eye Movement Data Transformation Technique that Preserves Temporal Information: A Demonstration in a Face Processing Task

**DOI:** 10.3390/s19102377

**Published:** 2019-05-23

**Authors:** Michał Król, Magdalena Ewa Król

**Affiliations:** 1 Department of Economics, The University of Manchester, Manchester M13 9PL, UK; 2 Wroclaw Faculty of Psychology, SWPS University of Social Sciences and Humanities, 53-238 Warszawa, Poland; mkrol1@swps.edu.pl

**Keywords:** eye tracking, scanpath comparison, dimensionality reduction, machine learning, autism, face perception

## Abstract

Existing research has shown that human eye-movement data conveys rich information about underlying mental processes, and that the latter may be inferred from the former. However, most related studies rely on spatial information about which different areas of visual stimuli were looked at, without considering the order in which this occurred. Although powerful algorithms for making pairwise comparisons between eye-movement sequences (scanpaths) exist, the problem is how to compare two groups of scanpaths, e.g., those registered with vs. without an experimental manipulation in place, rather than individual scanpaths. Here, we propose that the problem might be solved by projecting a scanpath similarity matrix, obtained via a pairwise comparison algorithm, to a lower-dimensional space (the comparison and dimensionality-reduction techniques we use are ScanMatch and t-SNE). The resulting distributions of low-dimensional vectors representing individual scanpaths can be statistically compared. To assess if the differences result from temporal scanpath features, we propose to statistically compare the cross-validated accuracies of two classifiers predicting group membership: (1) based exclusively on spatial metrics; (2) based additionally on the obtained scanpath representation vectors. To illustrate, we compare autistic vs. typically-developing individuals looking at human faces during a lab experiment and find significant differences in temporal scanpath features.

## 1. Introduction

It has been long established that human eye movement behavior is determined not only by the properties of the objects that are looked at, but also by factors related to the observer, i.e., that they are subject to ‘top-down’ influences independent of the ‘bottom-up’, stimulus-driven effects [1]. As a result, eye movement measurements recorded with eye-tracking camera devices, typically head-mounted or attached underneath computer screens, can be used to learn many things about the observer. For instance, they can help predict the viewer’s expertise [2] or cognitive capacity [3]. 

At the same time, given the complexity of eye data, many problems, such as predicting the task or purpose of the observer examining the stimulus, could only be solved with the use of advanced machine learning techniques [4]. Furthermore, even state-of-the-art analyses of this type are typically based on spatial but not temporal eye movement information, i.e., on which parts of an image were looked at, but not on when and in what order this has occurred. 

Nevertheless, two classes of eye movement modeling techniques exist which challenge this trend. The first one comprises probabilistic methods based on constructing, for an individual eye movement sequence (scanpath), a vector of metrics encoding the likelihoods of attention transferring between given ‘areas-of-interest’ (AOIs, i.e., any parts of the image of interest to the researcher). An example of this approach is the ‘Scanpath Successor Representation’ technique [5], successfully adapted to eye movement modeling [6], and recently applied, by the authors of this article, to the task of predicting decision bias from eye movements [7]. Other related approaches are based on modeling attention transitions via hidden Markov models [8]. Nevertheless, an analysis based on transition likelihoods may, in some contexts, be subject to certain drawbacks. In particular, a researcher might want to test if two sets of scanpaths, e.g., those registered with vs. without an experimental manipulation in place, or associated with two different groups of participants, have significantly different temporal features. However, certain differences might be more of interest, or more significant, than others. For instance, in the context of face perception (a major area of eye-tracking research), suppose that two scanpaths subject to comparison entail looking at the mouth and the left eye, respectively, at a given time. Such a difference is then likely to be more significant than if the two scanpaths entailed looking at the right vs. the left eye. In other words, although data-driven approaches are welcome when prediction is the ultimate aim, when seeking to compare scanpath sets one might want to specify a priori certain relationships between AOIs to focus the comparison on features of particular interest. For techniques based on transition likelihoods, this is not possible.

Fortunately, the other type of eye movement modeling techniques accounting for temporal information, namely those based on pairwise comparisons between scanpaths, can address the above limitation. In particular, the ScanMatch method [9] is an implementation of the Needleman–Wunsch algorithm [10] for the purpose of analyzing eye data. It is based on finding the optimal alignment between the two compared scanpaths, before producing a similarity score based on the mismatch between aligned scanpaths and the transformations required to align them. Crucially, in doing so, the algorithm takes into account a preset ‘substitution matrix’ which contains scores to be assigned to alignments between given AOI pairs—based either on spatial or semantic relationships between the AOIs. Thus, the distinguishing feature of ScanMatch is the ability to quantify all of the different types of scanpath characteristics: spatial, temporal (viewing duration), sequential (viewing order), and semantic [11]. 

Despite those advantages, a significant barrier in the use of ScanMatch in some contexts is that it assigns a similarity score to a given pair of scanpaths but cannot be used to evaluate individual scanpaths. Thus, we can use it to determine how different two scanpaths are from each other, but not if two groups of scanpaths (associated with different levels of an experimental factor) are significantly different. In theory, one can check if the within-group similarities are smaller than the between-group ones, but due to the lack of independence between the resulting similarity score observations, statistical testing of such a comparison is problematic. The purpose of the current paper is, therefore, to present a simple technique which can, nevertheless, make it possible to use the powerful pairwise scanpath comparison algorithms to compare groups of scanpaths. 

The essence of the proposed technique is first to compute the scanpath similarity matrix via pairwise comparisons between all scanpaths. Then, a dimensionality-reduction algorithm is used to map the scanpaths onto a low-dimensional space in such a way that their relative positioning therein reflects the corresponding pairwise similarity scores. The exact method we use in our demonstration is t-distributed stochastic neighbor embedding (t-SNE) [12], as it allows for a similarity matrix to be input directly at stage two of the algorithm, without providing it with the set of original high-dimensional vectors (which, in this case, does not exist). In fact, t-SNE has already been used in similar circumstances, e.g., to analyze similarities between words [13]. In the last step of the procedure, we propose to run a non-parametric test, such as the two-sample Cramer–von Mises test [14], to compare the distributions of dimension-reduced vectors corresponding to the two groups of scanpaths. If the low-dimensional projections of the two sets of scanpaths are found to be systematically different, then this suggests that so too are the original scanpaths. 

In order to demonstrate the proposed approach, we compare the scanpaths of subjects with vs. without autism spectrum disorder (ASD) recorded while examining images of faces in a number of facial processing tasks. Autism is a developmental disorder affecting social and communicative functioning and related to repetitive, limited patterns of behavior and interests. Previous studies revealed differences in eye movement patterns between ASD and typically developing individuals, especially with regard to social and facial stimuli ([15,16]) and particularly in terms of less attention being allocated to the eyes in the ASD population [17] (relatedly, behavioral studies have pointed to differences in visual short-term memory use, see [18]). However, these results rely on purely spatial analyses, e.g., using the iMAP toolbox [19], and less is known about whether or not the temporal dynamics of gaze in autism is also different. Furthermore, existing studies typically use facial images with cropped peripheral regions (i.e., without hair, neck, etc.) and such that the locations of the key facial regions are the same across images. This facilitates the analysis but can be seen as somewhat unrealistic. Aiming to demonstrate our proposed technique in this context, we used images of the entire head and a variety of faces with varying key region locations. To track the resulting changes in AOI locations across images, we applied a state-of-the-art facial features landmark detection algorithm [20]. After collecting the eye data of ASD subjects and typically developing (TD) controls who completed a variety of face processing tasks in two experiments, we processed the obtained scanpaths according to the procedure described above. 

We found a significant difference between the distributions of the dimension-reduced scanpath representation points corresponding to scanpaths of ASD vs. TD subjects. To further test if the result is indeed driven by temporal differences, we also compared the performance of two gradient boosted decision tree classifiers based on the LightGBM framework [21]. Both were trained to predict if a given scanpath was carried out (during a single two-second exposure of an image) by an ASD or a TD subject. However, one classifier was trained using spatial eye movement information alone, while the other included the dimension-reduced scanpath representation coordinates encoding temporal information in the set of features. It turned out that including temporal information in the proposed form in the feature set significantly increases the classifier’s accuracy. 

Overall, these results suggest that the proposed technique could be used in other problems in which one needs to compare two sets of eye movement sequences in terms of their temporal features. 

## 2. Materials and Methods

### 2.1. Subjects

We recruited 21 subjects with ASD (including 2 females) and 23 typically developing age- and IQ-matched subjects (5 females), who took part in a series of eye-tracking studies in the laboratory. The age range was 11–29 years in the ASD group (mean = 16.27) and 10–21 in the TD group (mean 16.31). All subjects had normal or corrected to normal vision and no neurological conditions. Intelligence was measured using the Wechsler Scales of Intelligence, and all subjects had full-scale IQs at least in the average range (>85), with mean IQ of 109.4 in the ASD group and 112.3 in the TD group. None of the TD subjects had a history of autism spectrum or other neurodevelopmental disorders. All participants in the ASD group had clinical diagnoses prior to their enrolment in the study, based on the criteria outlined in the World Health Organization International Classification of Diseases (ICD-10). All diagnoses were additionally confirmed by our team with the Autism Diagnostic Observation Schedule (ADOS 2), which is a standardized, validated instrument for the assessment of autism spectrum conditions, consisting of structured and semi-structured tasks that probe various aspects of social and communicative behaviors. The study was approved by the local Faculty Research Ethics Committee of the University of Social Sciences and Humanities in Wrocław, where it was conducted; written consent was obtained either from the adult subjects themselves or, in case of underage subjects, from their guardians in addition to oral consent from the underage subjects. 

### 2.2. Stimuli and Design

We used color photographs of 60 (half of them female, half of them male, an equal number of faces from each of the three age groups: young, middle-aged, older) faces from the FACES Database [22]. Each face in the database is presented with two versions of each of the same six emotional expressions (neutrality, anger, sadness, disgust, fear, and happiness). Each face was shown to each subject only once and with only one, randomly chosen expression, to avoid the familiarity effect from the repetition of the same face and potential interactions between specific faces and emotions. Face stimuli were randomly assigned to tasks (see the Procedure subsection) and presented in random order for each subject.

### 2.3. Procedure

Subjects were seated 65 cm in front of the screen of a 15” Dell Precision M4800 workstation (screen resolution 1280 × 720), with their heads placed in a chinrest. Their eye movements were recorded using a remote eye-tracking device SMI RED250 Mobile, with a sampling rate of 250 Hz and gaze position accuracy of 0.4°. The experimental software was programmed in C#. Prior to the commencement of the study, subjects completed a 5-point calibration and 4-point validation procedure. Additionally, eye data quality was periodically checked by the experimental software, which prompted subject repositioning when required. All experimental trials were preceded by a fixation cross displayed at the center of the screen for 500 ms, followed by the facial image displayed centrally for 2000 ms, and finally a blank screen or an appropriate task question prompt (see below) displayed for 2000 ms. 

In each trial, subjects completed one of five face perception tasks: (1) guessing the emotion expressed by the face; (2) judging which of two facial features: one of the brows or the lips, were wider; (3) freely viewing the face without any requirements or questions; (4) estimating the age of the person shown; and (5) judging whether or not the person appeared “nice”. The tasks were split into two experiments, with Experiment 1 featuring tasks 1–3, and Experiment 2 including tasks 4–5 (both experiments involved the same set of subjects described above). The tasks were carried out in blocks of 12 trials each, with the freeview task (3) block shown first in Experiment 1, and the order of the other blocks in each experiment being randomized, together with the ordering of individual faces within each block. Due to the fact that recruiting ASD subjects and validating their diagnoses is extremely costly, eye data from Experiment 1 was also used in a separate paper investigating information extraction by ASD subjects [23]. Here, we use pooled data from both experiments, comprising 5 × 12 = 60 trials per subject.

### 2.4. Data Analysis

The raw data is made up of sequences, one for each subject, and separately for the left and the right eye, of X-Y eye positions (in pixels, within the range determined by the screen resolution), at a sampling rate of 250 per second. As is the usual practice, the first step is to detect eye movement “events”, particularly eye fixations, defined as a pause of eye movement at a given point of the visual field. To this end, we used the SMI event detector high-speed detection algorithm with default settings (required fixation duration of 60 ms and a velocity threshold of 50 ^0^/s). Thus, we obtained a sequence of eye fixations for each subject/eye, each fixation characterized by its starting time, duration, and X-Y position. 

**Figure 1:**
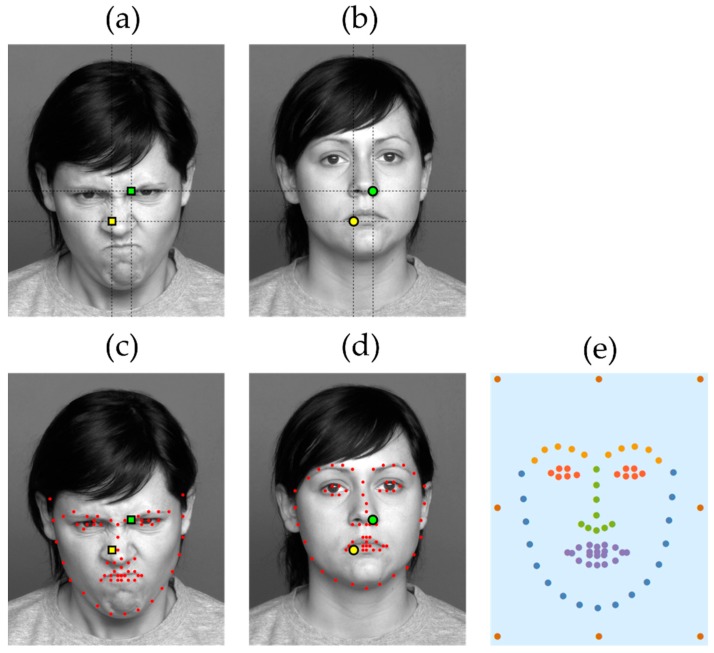
Despite having the same X-Y locations, the fixations indicated by yellow/green circles in the (**a**) image are aimed at different facial features than the corresponding yellow/green square-indicated fixations in the (**b**) image. To address this, we computed the locations of 68 key facial feature points (red dots in images **c** and **d**) and assigned each fixation to its nearest feature point (AOI; area-of-interest). The average key point locations across all face images are shown in panel (**e**) which also illustrates how the points were grouped into the face outline (blue), mouth (violet), nose (green), eyes (red), eyebrows (orange), and exterior frame points (brown).

The next step is to define the areas-of-interest in order to then assign the fixations to them. Here, the principal difficulty is that, as illustrated in Figure 1a–b, locations of key facial features may vary even across images of the same person expressing different emotions (and tend to vary even more across different people). Thus, we cannot simply split the image into tiles, as is often done in eye-tracking research, and take each tile as a distinct AOI. Doing so would result in fixations directed at different facial features being potentially assigned to the same AOI. 

To solve this problem, we used the recently developed machine learning facial features landmark detection algorithm [20] trained on a large dataset of 120,000 examples (we used the Wolfram Mathematica 11.3 implementation of the technique). For any given face image, the algorithm finds the locations of 68 key points corresponding to various features of the face (eyebrows, eyes, nose, mouth, and the overall outline of the face, see Figure 1c–d). Thus, for each fixation that occurred while a face image was shown, we calculated the distance between its position and that of each of the key feature points, as well as 8 additional equidistant points along a rectangular frame enclosing the face. We selected the point that the fixation was closest to, each of which constituted a separate AOI, encoded with one of 68 + 8 = 76 distinct characters (the additional 8 points were included to capture ‘stray’ fixations that were far away from the face—a relatively frequent occurrence among ASD subjects in particular). 

With each fixation assigned to an AOI, we then followed the ScanMatch procedure [9] in every detail (although other pairwise scanpath comparison techniques may be used instead). In particular, we needed to convert each sequence of fixations that occurred while a given face image was shown, a separate one for each eye, into a “scanpath” string describing the ordering and duration of AOI visits. To this end, for each subsequent fixation, the character corresponding to the AOI it was assigned to was repeated a number of times equal to the fixation duration divided by 50 ms, rounded to the nearest integer. The character strings obtained for the individual fixations were then combined in the order in which those fixations occurred, giving a scanpath representation encoding the fixation durations, one for each subject/trial/eye. Note that those trials in which no transitions between AOIs occurred at either eye were excluded from further analysis. Furthermore, the reason why we used binocular data (keeping the data corresponding to each eye separate), was that there was a possibility of a slight offset, with each eye showing a tendency to be directed slightly further towards its side of the face.

In the next step of the ScanMatch procedure, we conducted all possible pairwise comparisons between individual scanpaths using the Needleman–Wunsch algorithm. The algorithm’s substitution matrix was parametrized so as to reflect both the spatial and the semantic relationships between the AOIs. In particular, we grouped the AOIs according to the categories assigned to them by the landmark detection algorithm: the face outline, mouth, nose, eyes, eyebrows, as well as the exterior frame points, as shown in Figure 1, panel e. If two distinct AOIs belonged to the same group, a substitution between them would result in a positive score inversely proportional to the average Euclidean distance between them across all facial images used in the study; in contrast, a substitution between two AOIs from different groups would result in a negative score directly proportional to the distance. The scores were further re-scaled to range between −1 and +1, i.e., a substitution between the closest two feature points from the same group would yield a score of +1, whereas one between the two most distant points belonging to different groups would yield −1. The algorithm would, therefore, find the optimal global alignment of the two scanpath strings, seeking to align pairs of fixations directed at neighboring AOIs from the same group, and scoring the similarity of aligned scanpaths accordingly. Simply put, the similarity score would be high for those scanpaths where similar (closely positioned and semantically related) regions of the face were visited at similar times. 

As an extra precaution, to safeguard against the similarity scores being biased by varying scanpath lengths, the scores were normalized by dividing each one by the length of the longest of the two compared sequences. For example, if one temporally binned scanpath sequence contained 30, and the other 40 AOI character identifiers, then the ScanMatch similarity score obtained for this pair of scanpaths was divided by 40 (the length of the globally aligned sequence). Otherwise, shorter temporally binned scanpaths might generally be classed as more similar (due to having fewer mismatched elements), which would particularly apply to the ASD participants. Specifically, the average length of a scanpath string per trial was 29.22 (SD = 10.18) for ASD subjects and 30.87 (SD = 10.99) for TD subjects, corresponding to about 1.5 s.

At this point, we proceeded to our proposed customization. Specifically, the scanpath similarity matrix obtained as above was fed into the t-Distributed Stochastic Neighbor Embedding algorithm [12]. In our demonstration, we used the MATLAB implementation of t-SNE, particularly its variant which takes a similarity matrix as input and can, therefore, be used to dimension-reduce datasets with items that are not vectors, e.g., words [13]. In addition to this highly useful feature, and compared with the more conventional principal component analysis, t-SNE tends to perform better on datasets that feature complex non-linear relationships [24], which is likely to be the case for eye data. Each scanpath is mapped onto a point in a two-dimensional space in a way that minimizes the Kullback–Leibler divergence between the distributions modeling the original vs. dimension-reduced data. Simply put, scanpaths that are similar (in the sense of ScanMatch) would tend to be assigned to neighboring points in the low-dimensional space. Thus, for each subject/trial, we obtained two sets of dimension-reduced scanpath representation X-Y coordinates (one for each eye). Note that, in our demonstration, we used the original MATLAB code provided by van der Maaten, but reasonable adjustments to algorithm settings, like increasing the number of training rounds, learning rate, or momentum, did not result in any qualitative changes to the results presented in the next section. The data and code used to obtain the results described below are available as supplementary information.

## 3. Results

**Figure 2:**
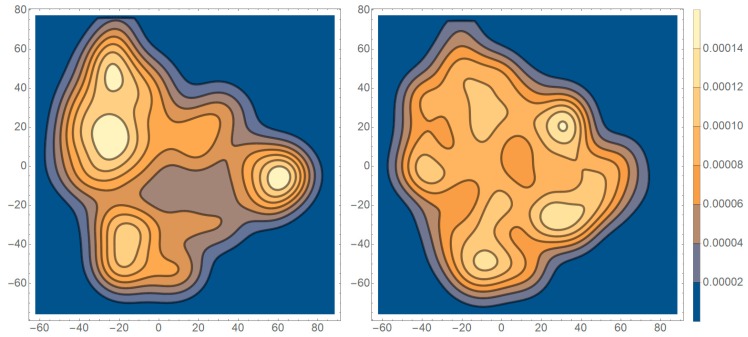
The smooth Gaussian kernel density estimates of the distribution of t-SNE dimension-reduced points obtained for typically developing (TD) (**left**) vs. autism spectrum disorder (ASD) (**right**) subjects (based on data from the right eye).

In the first instance, we wanted to test if the distributions of the t-SNE dimension-reduced points obtained for ASD vs. TD subjects, depicted in Figure 2, were significantly different. With this in mind, we used the non-parametric Cramer test [14], implemented via R [25], based on 1000 Monte-Carlo permutation resampling replicates. For t-SNE points corresponding to the left eye, we obtained a test statistic = 358.84, critical value = 61.59, and p < 0.001, making the difference statistically significant at an alpha-level of 0.05. A similar result was obtained for the right eye (test statistic = 205.25, critical value = 62.33, and p < 0.001). This suggests that the ASD and TD subjects’ scanpath representation points were distributed differently, which in turn indicates a likely difference between the original sets of scanpaths.

Nevertheless, one could argue that the observed differences are a consequence of spatial rather than both spatial and temporal differences. As has already been mentioned, there is a known tendency for ASD individuals to look more at the mouth relative to the eyes. To illustrate the problem, suppose that this observed difference is caused by TD subjects always looking at the mouth first and then at the eyes, whereas ASD subjects look at the mouth first and then only sometimes transfer their attention to the eyes. Even though the order of looking at the two facial regions is the same across the groups (i.e., there is no meaningful temporal difference per se), the ScanMatch algorithm would still detect a mismatch between the respective scanpaths, potentially leading to the statistically significant differences observed above. The question is then: once we have accounted for the spatial differences, is there also a difference in the temporal aspect? For instance, it might be the case that even those ASD subjects who look at both the eyes and the mouth (i.e., exhibit similar spatial scanpath tendencies to the TD group), will tend to do so in a different order, e.g., they might be more likely to look at the mouth first, whereas TD subjects might be more likely to first look at the eyes. If this were the case, adding the dimension-reduced scanpath representation scores to variables characterizing exclusively the scanpaths’ spatial properties should allow for improved discrimination between ASD vs. TD subjects’ scanpaths.

To test this idea, for each subject/trial/eye, we removed the temporal information from the associated scanpath by randomly permuting the scanpath string characters. Then, we used the same combination of ScanMatch and t-SNE as above to compute analogous, dimension-reduced scanpath representation scores that could now reflect only the frequencies of AOI hits but not the order in which the AOIs were looked at. Thus, for each facial image exposure, we obtained a collection of four numbers representing exclusively the spatial features of the accompanying scanpath (two for each eye), adding to the previously calculated four numbers similarly representing its spatial *as well as temporal* features.

We then carried out a 5-by-2 repeated cross-validation test [26] to compare two classifier models: (1) predicting the ASD vs. TD group membership based on the four spatial information feature variables alone; and (2) making the same prediction based on both spatial and temporal variables. In both cases, we used gradient boosted decision tree classifiers based on the LightGBM framework [21], trained using Wolfram Mathematica 11.3 under default settings: a maximum of 50 boosting rounds, maximum tree-depth set to 6, and automatically chosen leaf sizes. We have not observed any significant changes to our results when manipulating these defaults. Thus, we have used a similar technique to the one that was previously used and proved effective for spatial eye movement data alone [4]. Note that, each time the dataset was split into two folds, we ensured that each subject’s data entered only one of them, and that both subject groups were evenly split across the folds. Additionally, we set the classifier class prior probabilities to 0.5.

Overall, the accuracy of the “spatial” model (M = 53.9% across the five iterations and folds) was significantly smaller than that of the “spatial + temporal” model, M = 55.5%, t(5) = −2.94, p = 0.032. Adding additional variables to the set of predictors, such as the face emotion, gender, age, or the type of task, did not cause a significant increase of prediction accuracy for either model. The confusion matrices of the two models are presented in Table 1. Due to relatively low accuracy rates, we also used a permutation test [27] to verify that the performance of both models was significantly above the chance level (in both cases, we obtained a p < 0.001, based on k = 1000 random permutations). For reference purposes, we also repeated the above 5-by-2 repeated cross-validation process using, as a benchmark for t-SNE and ScanMatch, a combination of analogous but more standard techniques: Kruskal’s non-metric multidimensional scaling and the Levenshtein distance. In this case, we found that the accuracy of the “spatial” model (M = 51.8%) was not significantly smaller than that of the “spatial + temporal” model, M = 51.9%, t(5) = −0.94, p = 0.387, which might indicate that the t-SNE and ScanMatch combination can more accurately capture spatio-temporal differences between groups of scanpaths. 

## 4. Discussion

The results indicate that there is a statistically significant difference between the face-scanning patterns of ASD vs. TD individuals, and that it lies not only in their scanpaths’ spatial properties (as shown by existing studies) but also in their temporal aspects. First, the dimension-reduced scanpath representation scores, computed by t-SNE to reflect the pairwise ScanMatch similarities between them, were distributed differently for ASD than for TD subjects, suggesting that the original scanpaths were also different across the two groups. Second, the cross-validated accuracy of a model predicting the group membership based on spatial information alone (elicited via ScanMatch/t-SNE from randomly permuted scanpaths) was lower than that of a model which also included temporal data. This further supports the existence of temporal differences between the two sets of scanpaths because, although ScanMatch potentially captures both spatial and temporal differences, adding information from the original, ordered scanpaths to that from their permuted variants allows for more accurate discrimination between ASD and TD subjects.

Although the reported accuracies may seem low, one should consider that the prediction is made on the basis of an extremely short exposure to a single facial image (i.e., based on approximately 1.5 s of eye fixation data). Additionally, ScanMatch might be superior in capturing temporal features at the expense of a less accurate and less direct encoding of the potentially highly diagnostic spatial properties of the scanpaths. At the same time, even extended psychometric tests designed for autistic traits screening, like the Autism Spectrum Quotient [28] which features 50 questions, achieve a high sensitivity only at the cost of a specificity of about 50% [29]. The only other study which attempted to predict ASD traits based on (spatial) eye data [30] reported very high accuracies, but each prediction was made based on aggregated data from a large number of facial image viewings by the given subject, with the feature set and other parameters optimized to maximize the accuracy from the resulting small set of final observations (additionally, the faces were normalized, cropped, and displayed repeatedly). 

In any case, the purpose here was not ASD diagnostics, but the statistical testing of temporal differences between TD and ASD individuals’ gaze patterns. The positive result in this respect suggests that the proposed technique could be used, more generally, to identify temporal differences between any two sets of scanpaths, not only between- but also potentially within-subjects. For example, one could examine, in a repeated-measures design, the effect of an intervention aimed at changing the order in which people seek out and process certain pieces of information. 

Despite this, the proposed customization has, naturally, its limitations. First, unlike conventional techniques like principal component analysis (PCA), our application of t-SNE does not provide us with a function that directly maps from the original space onto the dimension-reduced one (if only because the original space does not, in the present case, exist). When using PCA, one can examine the properties of the said mapping to see how specific features of the original variables (here, scanpaths) determine the dimension-reduced scores, and hence how discriminative these features are with respect to the two sets of scanpaths (we did so in our previous work [7] using PCA combined with the Scanpath Successor Representation model). In our case, however, we know essentially that the two sets of scanpaths are temporally different, but not how different they are and what specific temporal aspects drive these differences. Additional descriptive analysis might, in general, be helpful to supplement and help interpret the basic statistical tests.

In addition, one limitation of t-SNE is that, in the absence of an explicit dimension reduction function, one can transform a given set of high-dimensional observations, but it is then impossible to add new, previously unseen high-dimensional points to the dimension-reduced set without re-running the entire analysis and possibly changing the locations of the existing points. This makes it somewhat impractical to use t-SNE as a data pre-processing technique when training prediction algorithms to be applied to previously unseen data and means that it is currently more popular as a data exploration technique. Nevertheless, when it comes to answering research questions rather than predictive applications, our results indicate that this technique may be useful in new, previously unexplored ways. In particular, it provides us with a workaround which makes it possible to use any one of a number of existing powerful pairwise scanpath comparison algorithms to compare not just pairs of individual scanpaths, but their entire sets, and to statistically test the significance of the obtained differences. We have chosen to illustrate this approach using ScanMatch, with its capacity for capturing spatial, temporal, and semantic properties of the scanpaths via the substitution matrix. However, depending on the context, it might be more appropriate to use other techniques such as the MultiMatch [31] or cross-recurrence analysis [32] (see [8] or [11] for an overview of available methods and their respective advantages). Rather than advocate a specific pairwise comparison method, our aim was to provide a way of using any of these methods to compare sets of scanpaths.

Furthermore, as we have seen here, training prediction algorithms that cannot be used with previously unseen data can still be helpful in answering interesting questions, such as whether or not ASD individuals display temporal eye movement atypicalities. Other potential applications might include learning how people solve certain tasks and what are their underlying beliefs by examining the similarity of the accompanying eye movement patterns to those registered while solving other tasks. For instance, in our previous work [33], we first manipulated the subject’s beliefs in two alternative ways prior to playing a two-player game, and trained a neural network predicting which of the two types of beliefs were induced based on spatial eye movement metrics obtained while subsequently playing. We then applied the trained classifier to instances of the game in which, instead of the manipulation, players received feedback about the counterpart’s past moves. By examining the classification results, we could then infer if the feedback made subjects process the available visual information similarly to when they were instilled with a certain type of beliefs. 

Nevertheless, a big advantage of facial stimuli is that they feature a fixed set of semantically defined AOIs (mouth, eyes, etc.). In more diverse images, e.g., of natural scenes, this is no longer the case, making it much more challenging to compare scanpaths accompanying many different stimuli. Even then, it might be possible to use computer vision techniques to identify various classes of objects in the images, such as people, vehicles, buildings, etc., and to examine if sets of scanpaths differ in terms of the order in which different object types are examined (for instance, individuals with autism might look at people only after they have examined non-social objects). In general, we believe that the technique proposed here could be used to compare eye movements across tasks, subject groups, and experimental conditions based on temporal rather than spatial features alone. Future research will determine its suitability in this respect.

## Figures and Tables

**Table 1:** The confusion matrices of the spatial model vs. the spatial + temporal model (aggregated across iterations and folds).

Spatial Model					Spatial + Temporal Model				
		predicted class					predicted class		
		TD	ASD				TD	ASD	
actual class	TD	3129 27.7%	2941 26.0%	53.7%	actual class	TD	3229 28.6%	2841 25.1%	53.7%
	ASD	2266 20.0%	2964 26.2%	46.3%		ASD	2189 19.4%	3041 26.9%	46.3%
		47.7%	52.3%				47.9%	52.1%	

## Supplementary Materials

The following are available online at https://www.mdpi.com/1424-8220/19/10/2377/s1: data; program code (Wolfram Mathematica 11.3 files).
